# Crystal structure of potassium orthoselenate(IV), K_2_SeO_3_

**DOI:** 10.1107/S2056989022005175

**Published:** 2022-05-17

**Authors:** Ralf Albrecht, Thomas Doert, Michael Ruck

**Affiliations:** a Faculty of Chemistry and Food Chemistry, Technische Universität Dresden, D-01062 Dresden, Germany; b Max Planck Institute for Chemical Physics of Solids, Nöthnitzer Str. 40, D-01187 Dresden, Germany; Vienna University of Technology, Austria

**Keywords:** crystal structure, hydro­flux, selen­ate(IV)

## Abstract

Crystal structure data for potassium orthoselenate(IV), K_2_SeO_3_, are reported for the first time. Colorless, block-shaped crystals were grown in a potassium hydro­flux, *i.e.* under ultra-alkaline conditions, within 10 h. K_2_SeO_3_ crystallizes isostructural with Na_2_SO_3_ and K_2_TeO_3_ in the trigonal space group *P*  with lattice parameters *a* = 6.1063 (4) Å and *c* = 6.9242 (4) Å at 100 (1) K. In the trigonal–pyramidal, *C*_3v_-symmetric [SeO_3_]^2–^ anion, the bond length is 1.687 (1) Å, and the bond angle is 103.0 (1)°. Two of the three unique potassium cations exhibit a coordination number of six, and the third a coordination number of nine.

## Abstract 2

Potassium orthoselenate(IV), K_2_SeO_3_, crystallizes isostructural with Na_2_SO_3_ and K_2_TeO_3_ in the trigonal space group *P* 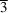 with lattice parameters *a* = 6.1063 (4) Å and *c* = 6.9242 (4) Å at 100 K.

## 1. Chemical context

Ternary alkali metal selenates(IV) are a long-known but poorly studied class of compounds. After the discovery of the first salts of selenic acid by Berzelius, comprehensive studies on these salts were not carried out until the beginning of the 1930s, when Janitzki reported the syntheses of sodium and potassium salts of selenic acid (Janitzki, 1932 ▸). Moreover, the composition and solubility of hydrates and anhydrates of these selenates(IV) were determined. However, only two crystal structures of ternary alkali metal selenates(IV) are known to date, *viz*. K_2_Se_2_O_5_ (Rider *et al.*, 1985 ▸) and Na_2_SeO_3_ (Helmholdt *et al.*, 1999 ▸; Wickleder, 2002 ▸). The latter compound was synthesized by annealing a mixture of Na_2_O and SeO_2_ at 773 K.

In this communication, we report on the synthesis and crystal structure of potassium orthoselenate(IV), K_2_SeO_3_. The title compound was synthesized using the hydro­flux approach, an ultra-alkaline reaction medium consisting of an approximately equimolar mixture of water and alkali metal hydroxide (Bugaris *et al.*, 2013 ▸; Chance *et al.*, 2013 ▸). Advantages of the hydro­flux method are the good solubility of oxides and hydroxides, the fast and simple reaction at moderate temperatures, and the formation of single-crystals suitable for X-ray diffraction. Moreover, the high hydroxide concentration within the hydro­flux reduces the activity of water, leading to the unexpected fact that water-sensitive products can be isolated, *e.g.* K_2_[Fe_2_O_3_(OH)_2_] (Albrecht *et al.*, 2019 ▸), Tl_3_IO (Albrecht *et al.*, 2020 ▸), or K_2_Te_3_ (Albrecht & Ruck, 2021 ▸).

## 2. Structural commentary

**Figure 1:**
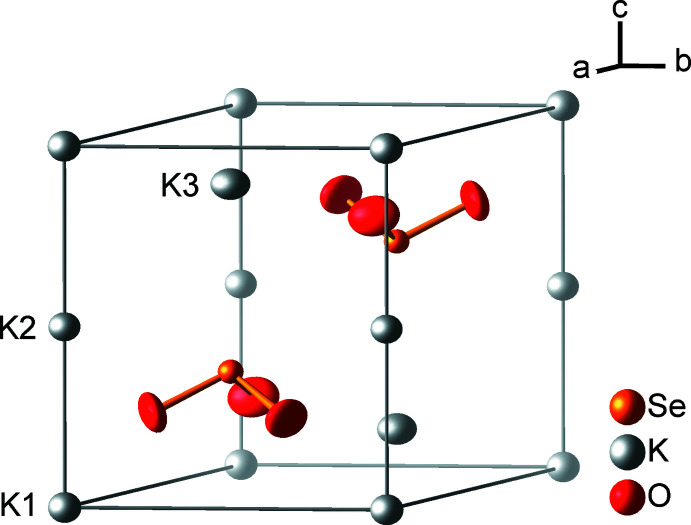
Crystal structure of K_2_SeO_3_ at 100 K, with displacement ellipsoids drawn at the 99% probability level; the unit cell is outlined.

**Figure 2:**
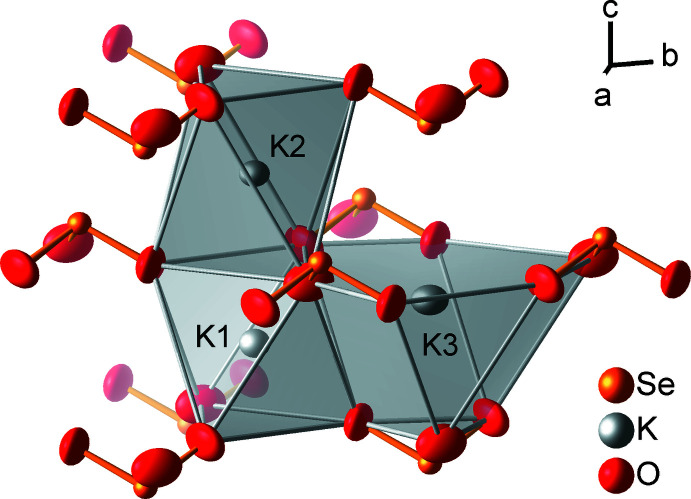
Coordination polyhedra of the potassium atoms, with displacement ellipsoids drawn at the 99% probability level.

Five atoms represent the asymmetric unit of K_2_SeO_3_, one selenium atom (site symmetry 3.., Wyckoff position 2*d*), three potassium atoms (K1: 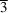 .., 1*a*; K2: 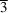 .., 1*b*; K3: 3.., 2*d*) and one oxygen atom (1, 6*g*). The unit cell of K_2_SeO_3_ is depicted in Fig. 1 ▸. The selenium atom is bound to three oxygen atoms with a Se—O bond length of 1.687 (1) Å and a bond angle O—Se—O of 103.0 (1)°. The pyramidal shape of the *C*_3v_-symmetric [SeO_3_]^2–^ anion can be attributed to the electron lone pair of the selenium(IV) atom. This oxidation state is supported by the bond-valence sum calculation (Brese & O’Keeffe, 1991 ▸) for selenium *ν*(Se) = ∑exp [(*R*_SeO_ – *d*_SeO_)/*b*)] = 3 · exp [(1.811 Å – 1.687 (1) Å) / 0.37 Å)] = 4.2 valence units. The potassium cations K1 and K2 are octa­hedrally coordinated by oxygen atoms with K—O distances of 2.631 (1) and 2.771 (1) Å, respectively. K3 has nine oxygen neighbors at distances of 2.792 (1), 3.020 (1) Å, and 3.474 (1) Å (Fig. 2 ▸).

**Figure 3:**
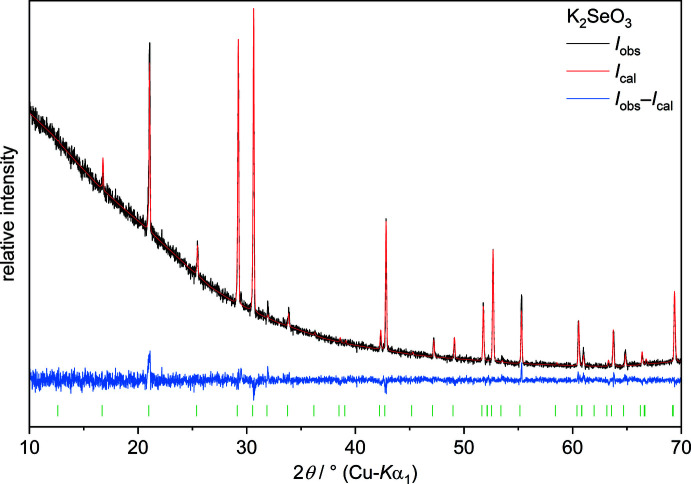
Powder X-ray diffractogram and Rietveld refinement of ground K_2_SeO_3_ crystals measured in a capillary at room temperature [*a* = 6.1114 (1) Å, *c* = 6.9938 (1) Å; *R*_p_ = 0.056, *wR*_p_ = 0.057, gof = 1.21].

It is noted that the X-ray powder diffraction pattern of ground K_2_SeO_3_ crystals (Fig. 3 ▸) differs significantly from previously published data (Hanawalt *et al.*, 1938 ▸; Klushina *et al.*, 1968 ▸).

## 3. Database survey

K_2_SeO_3_ crystallizes isostructural with Na_2_SO_3_ (Zachariasen & Buckley, 1931 ▸; Larsson & Kierkegaard, 1969 ▸) and K_2_TeO_3_ (Andersen *et al.*, 1989 ▸). On a more general level, the structure of K_2_SeO_3_ can be related to the Ni_2_In type in space group *P*6_3_/*mmc* (Laves & Wallbaum, 1942 ▸), with the K^+^ ions on the Ni positions and [SeO_3_]^2–^ anions occupying the positions of the In atoms. The orientation of the selenate(IV) groups is responsible for the symmetry reduction to *P* 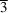 ; the higher pseudo-symmetry is mirrored in the respective twin laws.

## 4. Synthesis and crystallization

**Figure 4:**
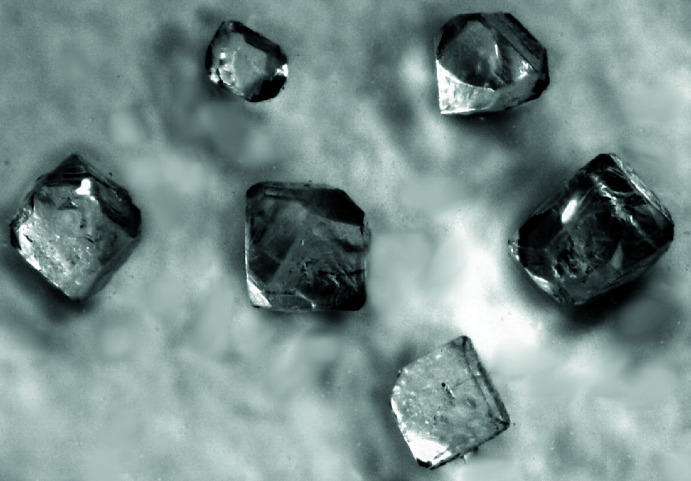
Photograph of K_2_SeO_3_ crystals.

**Figure 5:**
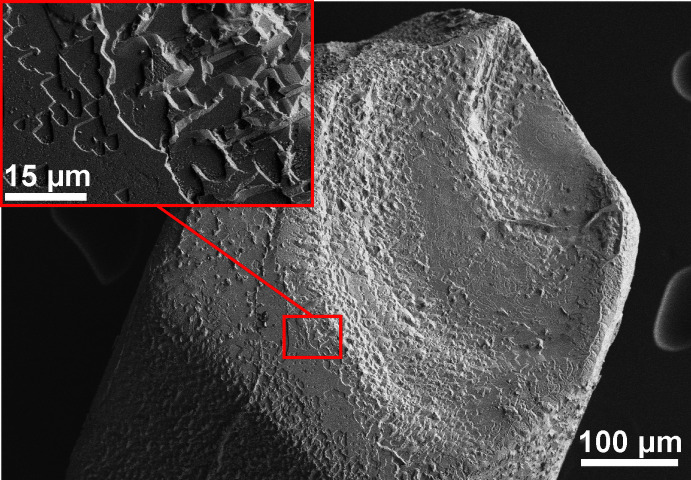
Scanning electron microscopy image after the washing process.

Potassium orthoselenate(IV), K_2_SeO_3_, was synthesized in a potassium hydroxide hydro­flux with a molar water-base ratio of 1.7. The reaction was carried out in a PTFE-lined 50 mL Berghof-type DAB-2 stainless steel autoclave to prevent evaporation of water. The starting material SeO_2_ (4 mmol, abcr, 99.8%) was dissolved in 3 ml of water before adding 6.3 g of KOH (Fischer Scientific, 86%). After closing the autoclave, the reaction mixture was heated to 473 K at a rate of 2 K min^−1^ and, after 8 h, cooled to room temperature at a rate of −1 K min^−1^. The solid reaction product was washed twice with 2 ml of methanol on a Schlenk frit under inert conditions to remove adherent hydro­flux. The colorless, block-shaped crystals of K_2_SeO_3_ (Fig. 4 ▸) dissolve readily in water, but dissolve in methanol a little slower than the hydro­flux. Scanning electron microscopy showed that the surface of the crystals was etched by the washing process (Fig. 5 ▸). Due to its hygroscopicity, the product was dried in dynamic vacuum and stored under argon. Pure K_2_SeO_3_ was obtained with a yield of about 50%. Energy-dispersive X-ray spectroscopy on selected crystals confirmed the chemical composition within the limits of the method.

For the Rietveld refinement, the program *JANA2006* was used (Petříček *et al.*, 2014 ▸). Scanning electron microscopy was performed using a SU8020 (Hitachi) with a triple detector system for secondary and low-energy backscattered electrons (*U*_a_ = 5 kV). The composition of selected single crystals was determined by semi-qu­anti­tative energy dispersive X-ray analysis (*U*_a_ = 15 kV) using a Silicon Drift Detector X–MaxN (Oxford Instruments). The data were processed applying the *AZtec* software package (Oxford Instruments, 2013 ▸).

## 5. Refinement

Crystal data, data collection and structure refinement details are summarized in Table 1 ▸. The investigated crystal was found to be a fourfold twin: twinning by merohedry plus twofold rotation along [001]. The crystal, thus, partially conserves the hexa­gonal (pseudo-)symmetry of the Ni_2_In type.

## Supplementary Material

Crystal structure: contains datablock(s) global, I. DOI: 10.1107/S2056989022005175/wm5645sup1.cif

Structure factors: contains datablock(s) I. DOI: 10.1107/S2056989022005175/wm5645Isup2.hkl

CCDC reference: 2172487

Additional supporting information:  crystallographic information; 3D view; checkCIF report

## Figures and Tables

**Table 1:** Experimental details

Crystal data	
Chemical formula	K_2_SeO_3_
*M*_r_	205.2
Crystal system, space group	Trigonal, *P* 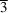
Temperature (K)	100
*a*, *c* (Å)	6.1063 (2), 6.9242 (4)
*V* (Å^3^)	223.59 (2)
*Z*	2
Radiation type	Mo *K*α
μ (mm^−1^)	10.11
Crystal size (mm)	0.05 × 0.05 × 0.02

Data collection	
Diffractometer	Bruker APEXII CCD
Absorption correction	Multi-scan (*SADABS*; Krause *et al.*, 2015 ▸)
*T*_min_, *T*_max_	0.539, 0.747
No. of measured, independent and observed [*I* > 3σ(*I*)] reflections	12526, 790, 785
*R*_int_	0.021
(sin θ/λ)_max_ (Å^−1^)	0.858

Refinement	
*R*[*F* > 3σ(*F*)], *wR*(*F*), *S*	0.009, 0.033, 1.05
No. of reflections	790
No. of parameters	24
Δρ_max_, Δρ_min_ (e Å^−3^)	0.77, −1.51

Computer programs: *APEX2* (Bruker, 2016 ▸), *SAINT* (Bruker, 2016 ▸), *SUPERFLIP* (Palatinus & Chapuis, 2007 ▸), *JANA2006* (Petříček *et al.*, 2014 ▸), *DIAMOND* (Brandenburg, 2021 ▸), and *publCIF* (Westrip 2010 ▸).

## supplementary crystallographic information

### Crystal data

**Table:**

K_2_SeO_3_	*D*_x_ = 3.047 Mg m^−^^3^
*M**_r_* = 205.2	Mo *K*α radiation, λ = 0.71073 Å
Trigonal, *P*3	Cell parameters from 7032 reflections
Hall symbol: -P 3	θ = 2.9–37.6°
*a* = 6.1063 (2) Å	µ = 10.11 mm^−^^1^
*c* = 6.9242 (4) Å	*T* = 100 K
*V* = 223.59 (2) Å^3^	Block, colourless
*Z* = 2	0.05 × 0.05 × 0.02 mm
*F*(000) = 192	

### Data collection

**Table:**

Bruker APEXII CCD diffractometer	790 independent reflections
Radiation source: X-ray tube	785 reflections with *I* > 3σ(*I*)
Graphite monochromator	*R*_int_ = 0.021
ω– and φ–scans	θ_max_ = 37.6°, θ_min_ = 2.9°
Absorption correction: multi-scan (*SADABS*; Krause *et al.*, 2015)	*h* = −10→10
*T*_min_ = 0.539, *T*_max_ = 0.747	*k* = −10→10
12526 measured reflections	*l* = −11→11

### Refinement

**Table:**

Refinement on *F*^2^	Primary atom site location: chargeflipping
*R*[*F* > 3σ(*F*)] = 0.009	Secondary atom site location: difference Fourier map
*wR*(*F*) = 0.033	Weighting scheme based on measured s.u.'s *w* = 1/(σ^2^(*I*) + 0.000576*I*^2^)
*S* = 1.05	(Δ/σ)_max_ = 0.001
790 reflections	Δρ_max_ = 0.77 e Å^−^^3^
24 parameters	Δρ_min_ = −1.51 e Å^−^^3^
0 restraints	Extinction correction: B-C type 1 Gaussian isotropic (Becker & Coppens, 1974)
0 constraints	Extinction coefficient: 570 (40)

### Fractional atomic coordinates and isotropic or equivalent isotropic displacement parameters (Å^2^)

**Table:**

	*x*	*y*	*z*	*U*_iso_*/*U*_eq_	
Se	0.666667	0.333333	0.338432 (15)	0.00495 (4)	
K1	0	0	0	0.00741 (8)	
K2	0	0	0.5	0.00764 (8)	
K3	0.333333	0.666667	0.14233 (5)	0.01091 (6)	
O	0.38608 (13)	0.25027 (14)	0.23422 (10)	0.0135 (2)	

### Atomic displacement parameters (Å^2^)

**Table:**

	*U*^11^	*U*^22^	*U*^33^	*U*^12^	*U*^13^	*U*^23^
Se	0.00527 (5)	0.00527 (5)	0.00432 (6)	0.00263 (3)	0	0
K1	0.00743 (9)	0.00743 (9)	0.00735 (14)	0.00372 (5)	0	0
K2	0.00816 (9)	0.00816 (9)	0.00658 (13)	0.00408 (5)	0	0
K3	0.01248 (8)	0.01248 (8)	0.00778 (10)	0.00624 (4)	0	0
O	0.0064 (3)	0.0208 (4)	0.0126 (3)	0.0062 (2)	−0.0022 (2)	0.0004 (2)

### Geometric parameters (Å, º)

**Table:**

Se—O	1.6865 (8)	K2—K3	4.3084 (4)
Se—O^i^	1.6865 (12)	K2—K3^xiii^	4.3084 (4)
Se—O^ii^	1.6865 (7)	K2—K3^xiv^	4.3084 (3)
K1—K2^iii^	3.4621 (4)	K2—K3^xv^	4.3084 (4)
K1—K2	3.4621 (4)	K2—O	2.7708 (7)
K1—K3^iv^	3.6606 (3)	K2—O^ix^	2.7708 (10)
K1—K3^v^	3.6606 (1)	K2—O^x^	2.7708 (8)
K1—K3	3.6606 (3)	K2—O^xiii^	2.7708 (7)
K1—K3^vi^	3.6606 (3)	K2—O^xvi^	2.7708 (10)
K1—K3^vii^	3.6606 (1)	K2—O^xvii^	2.7708 (8)
K1—K3^viii^	3.6606 (3)	K3—K3^vii^	4.0391 (4)
K1—O	2.6307 (7)	K3—K3^viii^	4.0391 (3)
K1—O^ix^	2.6307 (10)	K3—K3^xviii^	4.0391 (4)
K1—O^x^	2.6307 (8)	K3—O	2.7915 (10)
K1—O^vi^	2.6307 (7)	K3—O^xix^	2.7915 (8)
K1—O^xi^	2.6307 (10)	K3—O^xx^	2.7915 (12)
K1—O^xii^	2.6307 (8)	K3—O^viii^	3.0203 (8)
K2—K3^iv^	4.3084 (4)	K3—O^xxi^	3.0203 (10)
K2—K3^v^	4.3084 (3)	K3—O^xii^	3.0203 (8)
			
O—Se—O^i^	103.03 (4)	O—K2—O^x^	80.69 (2)
O—Se—O^ii^	103.03 (4)	O—K2—O^xiii^	180
O^i^—Se—O^ii^	103.03 (5)	O—K2—O^xvi^	99.31 (2)
O—K1—O^ix^	85.98 (3)	O—K2—O^xvii^	99.31 (2)
O—K1—O^x^	85.98 (2)	O^ix^—K2—O^x^	80.69 (3)
O—K1—O^vi^	180	O^ix^—K2—O^xiii^	99.31 (2)
O—K1—O^xi^	94.02 (3)	O^ix^—K2—O^xvi^	180
O—K1—O^xii^	94.02 (2)	O^ix^—K2—O^xvii^	99.31 (3)
O^ix^—K1—O^x^	85.98 (3)	O^x^—K2—O^xiii^	99.31 (2)
O^ix^—K1—O^vi^	94.02 (3)	O^x^—K2—O^xvi^	99.31 (3)
O^ix^—K1—O^xi^	180	O^x^—K2—O^xvii^	180
O^ix^—K1—O^xii^	94.02 (3)	O^xiii^—K2—O^xvi^	80.69 (2)
O^x^—K1—O^vi^	94.02 (2)	O^xiii^—K2—O^xvii^	80.69 (2)
O^x^—K1—O^xi^	94.02 (3)	O^xvi^—K2—O^xvii^	80.69 (3)
O^x^—K1—O^xii^	180	O—K3—O^xix^	114.97 (3)
O^vi^—K1—O^xi^	85.98 (3)	O—K3—O^xx^	114.97 (2)
O^vi^—K1—O^xii^	85.98 (2)	O—K3—O^viii^	92.04 (2)
O^xi^—K1—O^xii^	85.98 (3)	O—K3—O^xxi^	132.80 (3)
O—K2—O^ix^	80.69 (2)	O—K3—O^xii^	82.84 (2)

Symmetry codes: (i) −*y*+1, *x*−*y*, *z*; (ii) −*x*+*y*+1, −*x*+1, *z*; (iii) *x*, *y*, *z*−1; (iv) *x*−1, *y*−1, *z*; (v) *x*, *y*−1, *z*; (vi) −*x*, −*y*, −*z*; (vii) −*x*, −*y*+1, −*z*; (viii) −*x*+1, −*y*+1, −*z*; (ix) −*y*, *x*−*y*, *z*; (x) −*x*+*y*, −*x*, *z*; (xi) *y*, −*x*+*y*, −*z*; (xii) *x*−*y*, *x*, −*z*; (xiii) −*x*, −*y*, −*z*+1; (xiv) −*x*, −*y*+1, −*z*+1; (xv) −*x*+1, −*y*+1, −*z*+1; (xvi) *y*, −*x*+*y*, −*z*+1; (xvii) *x*−*y*, *x*, −*z*+1; (xviii) −*x*+1, −*y*+2, −*z*; (xix) −*y*+1, *x*−*y*+1, *z*; (xx) −*x*+*y*, −*x*+1, *z*; (xxi) *y*, −*x*+*y*+1, −*z*.
